# Adult Granulosa Cell Tumor With Low‐Level Amplifications of *JAK2*, *ERBB3*, *CDK4*, and *MDM2*: A Rare Testicular Tumor

**DOI:** 10.1155/criu/4462826

**Published:** 2026-07-28

**Authors:** Christopher Moran, Catherine Skibiel, Hadi Shojaei, Jeffrey J. Tomaszewski, Tina Bocker Edmonston, Veniamin Barshay, Ruth Birbe

**Affiliations:** ^1^ Department of Internal Medicine, Rutgers Health Robert Wood Johnson Medical School, New Brunswick, New Jersey, USA; ^2^ Department of Pathology and Laboratory Medicine, Cooper University Health Care, Camden, New Jersey, USA, lhsc.on.ca; ^3^ Cooper Medical School of Rowan University, Camden, New Jersey, USA, rowan.edu; ^4^ Division of Urology, Department of Surgery, Cooper University Health Care, Camden, New Jersey, USA; ^5^ Department of Radiology, Cooper University Health Care, Camden, New Jersey, USA

## Abstract

Testicular adult granulosa cell tumors (AGCTs) are exceptionally rare neoplasms that are thought to be the male counterpart of ovarian granulosa cell tumors (GCT). Here, we present a 50‐year‐old African American male who initially presented with right testicular pain and swelling, with ultrasound revealing a 2.0‐cm heterogeneous hypoechoic lesion along the upper pole/interpolar right testicle with internal vascularity. Microscopic examination was consistent with AGCT, demonstrating monomorphic cells with elongated nuclei containing numerous small grooves, resembling ovarian granulosa cells, and scant cytoplasm arranged in trabeculae and sheets. Immunohistochemical stains of the tumor were positive for inhibin, vimentin, and weakly positive for calretinin, S100, and Ki67 (10%). Next Generation Sequencing (NGS) showed low‐level amplifications of *JAK2*, *ERBB3*, *CDK4*, and *MDM2*. No gene fusions or mutations were identified. The patient underwent a radical orchiectomy and remains alive and disease‐free at 42 months following diagnosis.

## 1. Introduction

Adult granulosa cell tumors (AGCTs) of the testis are the male counterpart of ovarian GCTs. AGCTs are rare neoplasms with an unknown incidence, with approximately 100 published cases to date [1]. In females, granulosa cells surround oocytes and produce reproductive hormones that support ovarian follicles and regulate normal metabolic processes. It is believed that some cells of the male gonadal stroma retain their embryological ability to differentiate into male or female stromal cells. This can allow traditionally female sex cord stromal cell tumors (e.g. granulosa cell tumors) to form in males [1]. The two major subtypes of ovarian and testicular GCTs are adult and juvenile. The juvenile GCT subtype occurs in patients less than 1 year of age, while the adult GCT subtype can occur any time thereafter [2]. There is very little data published to date on testicular AGCTs, and we seek to contribute to the current literature with this case report.

## 2. Case Description

**Figure 1:**
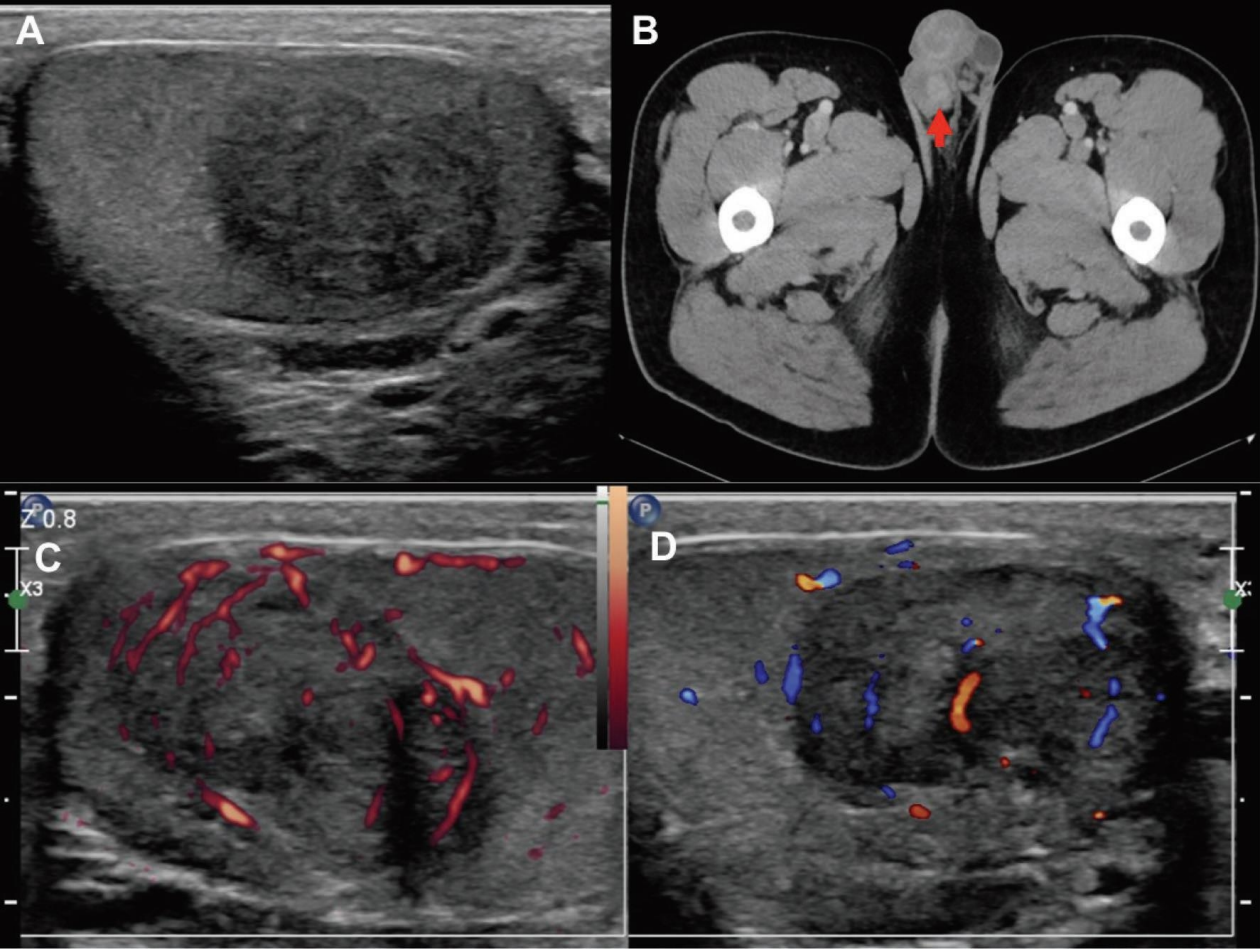
(A) Ultrasound of the right testis demonstrates a heterogeneous hypoechoic 1.3 × 2 × 2 cm lesion along the upper to interpolar region. (B) Non‐contrast CT showing a right testis mass red arrow. (C and D) Color Doppler.

A 50‐year‐old African American male presented to the Urology office with a month‐long history of right orchialgia and swelling. The patient also reported a lump on his right testicle over the prior 1–2 years, with a history of a remote right testicular cyst noted on ultrasound 7 years prior. The patient otherwise reported feeling well and denied dysuria, hematuria, prior urinary tract infection, prostatitis, epididymitis, or erectile dysfunction. Physical exam was remarkable for an uncircumcised, normal‐appearing penis and firm, slightly tender right testicular mass. An ultrasound of the right testicle revealed a 1.3 × 2.0 × 2.0 cm lesion along the upper to interpolar region with internal vascularity, concerning for a malignant germ cell tumor (Figure 1). The patient underwent a right radical inguinal orchiectomy without complication.

**Figure 2:**
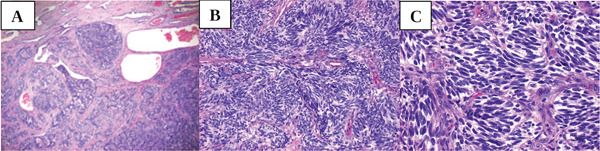
H&E stains of (A) GCT in rete testis 40×, (B) GCT 200×, (C) GCT 400×. The cells have elongated nuclei with small grooves, scant cytoplasm, and are arranged in trabeculae and sheets.

Macroscopically, the tumor was bright yellow, well‐circumscribed, and measured 1.7 × 1.2 × 1 cm. The tumor was limited to the testis with rete testis invasion. Histologically, the tumor was composed of monomorphic cells arranged in palisading trabeculae and sheets, with round to ovoid elongated nuclei, prominent longitudinal nuclear grooves, indistinct cell borders, and scant eosinophilic cytoplasm (Figure 2). The mitotic count was less than 1 per 10 high‐power fields. There was no evidence of necrosis or lymphovascular invasion.

**Figure 3:**
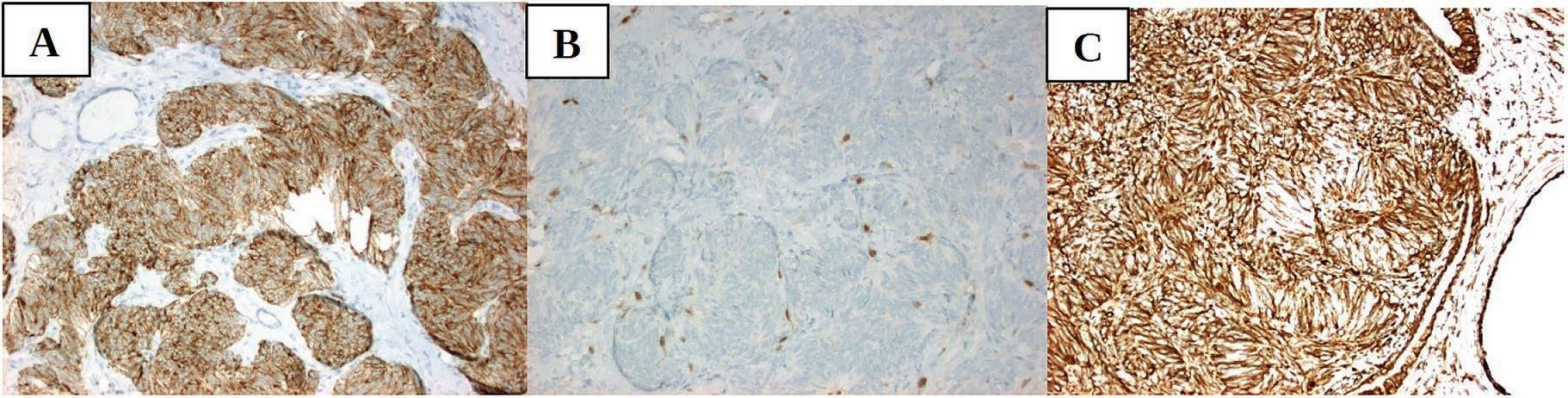
Immunohistochemical stains of the GCT. (A) Inhibin 200×. (B) Calretinin 200× (weak/patchy). (C) Vimentin 200×.

Immunohistochemical stains were positive for inhibin, vimentin, and weakly positive for calretinin, and S100 (Figure 3). The stains were negative for SALL4, beta‐catenin, pancytokeratin (AE1/AE3), cytokeratin OSCAR, CAM5.2, synaptophysin, chromogranin, GFAP, NSE, CD10, smooth muscle actin, desmin, CD99, and WT1. Beta‐catenin showed non‐specific cytoplasmic staining, but no nuclear staining. The Ki67 proliferation index was up to 10%. Reticulin stain highlighted large tumor cell groups. The final pathologic diagnosis was a Granulosa Cell Tumor, Adult Type. The pathologic stage classification was pT1a, pN and pM not assigned, based on the American Joint Commission on Cancer 8th Edition. Next Generation Sequencing (NGS) using the TST170 assay on a NextSeq 550 instrument (Illumina, San Diego, CA) showed low‐level amplifications of *JAK2*, *ERBB3*, *CDK4*, and *MDM2*. Follow‐up CT scans at 6 and 23 months showed no evidence of metastatic disease in the chest, abdomen, and pelvis. The patient is alive and disease‐free at 42 months following diagnosis.

## 3. Discussion

Granulosa cell tumors are derived from elements of sex cord stroma and are divided into adult and juvenile (occurring in the first year of life) subtypes [2]. Testicular AGCT is an exceedingly rare neoplasm with approximately 100 cases reported to date. The first case was reported by Laskowski in 1952, and the number of published cases since then has been increasing. This is likely due to improved awareness and more accurate classification of rare testicular neoplasms combined with increased ease of publication with the advent of many new internet‐based scientific journals [1].

The 50‐year‐old man described in this case falls within the expected age range of presentation for AGCTs, which is 26–55 years, with a median age of 44 years based on published reports. The oldest and youngest reported patients were 87 and 12 years old, respectively [1]. Patients typically present with a painless testicular mass or swelling, and up to 25% present with endocrine manifestations such as decreased libido and gynecomastia due to abnormal tumor sex hormone production. [3]. This patient experienced testicular pain and swelling but no endocrine manifestations. The size of testicular AGCT ranges from microscopic to up to 13.0 cm, with a median size of 3.2 cm. This tumor was slightly smaller at 1.7 cm. Grossly, AGCTs appear yellow/gray in color and are well circumscribed with a solid or partially cystic appearance. On ultrasound examination, AGCTs appear as a hypoechoic, well‐circumscribed intraparenchymal testicular mass [1]. Testis‐sparing surgery may be considered given the reported 13.5% incidence of malignancy. Although most AGCTs are benign, slow‐growing neoplasms, most are removed via radical orchiectomy due to the difficulty of distinguishing benign and malignant testicular tumors in a clinical setting [1]. Our patient underwent a right radical orchiectomy without adjuvant therapy because the tumor was confined to the testis without evidence of metastasis on staging CT scans. Features associated with malignancy reported by Jimenez‐Quintero et al. include large tumor size (greater than 7 cm), lymphovascular invasion, hemorrhage/necrosis, and increased mitotic activity [4]. Our patient presented with a right‐sided tumor, while a slight majority (54.3%) of reported tumors are left‐sided [1]. However, the sample size of published data is likely insufficient to make any strong statements regarding the usual laterality of these rare neoplasms.

The histopathologic pattern of AGCTs can be described as monomorphic cells resembling ovarian granulosa cells with scant cytoplasm arranged in trabeculae and sheets. Nuclei are elongated containing numerous small grooves. Call‐Exner bodies are a pathognomonic feature and are described as a microfollicular pattern of small cystic spaces containing hyalinized basement membrane or eosinophilic material, although none were found in this case. Immunohistochemically, AGCTs are positive for vimentin, inhibin, and calretinin and negative for pancytokeratin and typical germ cell tumor‐specific markers like Oct 3/4 and placental alkaline phosphatase [1]. Our patient’s tumor stained positive for inhibin, vimentin, and weakly positive for calretinin, S100, and Ki67. It was negative for beta‐catenin, Sall4, and cytokeratins.

Testicular AGCTs are molecularly heterogeneous and rarely harbor *FOXL2* mutations (approximately 10%) while 70% of cases show a single copy loss of 22q [5]. This contrasts molecular findings in metastatic ovarian AGCTs with *FOXL2* c.7558C>T (p.C134W) mutations reported in 92% of cases and 22q loss in 42% of tumors [5]. The *FOXL2* c.7558C>T (p.C134W) activating mutation is thought to lead to uncontrolled granulosa cell differentiation and growth [5]. We found no evidence of the common p.C134W missense mutation or of any other mutations sequencing all exons of the *FOXL2* gene. Interestingly, NGS of this specimen showed low‐level amplification of *MDM2*, *CDK4*, *ERBB3*, and *JAK2*. Amplification of chromosome 12q, where *CDK4*, *MDM2*, and *ERBB3* are located, has been reported in a variety of tumors including liposarcoma [6, 7], neuroblastoma [8], and some salivary gland tumors [9]. The *CDK4* gene encodes a catalytic subunit of the protein kinase complex that is critical for cell cycle progression through the G1‐S phase and leads to the constitutive activation of the cell cycle. Mutations in *CDK4* are implicated in the tumorigenesis of many cancers [10, 11]. Importantly, its expression is ubiquitous in ovarian tissue but less so in the testis. Skowron et al. found that CDK4 is highly expressed in adult germ cell tumor cell lines, and the tumors are highly susceptible to treatment with *CDK4*/*6* inhibitors in vitro, presenting a potentially important therapeutic option for patients with advanced disease and relapse [11]. Necchi et al. reported similar implications in testicular germ cell tumors, demonstrating a potentially significant opportunity for treatment with *CDK4* inhibitors. However, data regarding *CDK4* in AGCTs remain limited. *MDM2* regulates apoptosis of cells via ubiquitin‐dependent proteasomal degradation of the tumor suppressor p53 and is a known regulator of apoptosis in several body tissues, including granulosa cells [12, 13]. While its prognostic relevance to ovarian AGCTs is still under investigation [10, 14], recent studies by Lobo et al. revealed that “*MDM2* overexpression may indicate a more aggressive tumor phenotype, with propensity for therapy resistance and recurrence” in testicular germ cell tumors. Moreover, Colecchia et al. found that *MDM2* and *CDK4* amplification was more likely to be found in malignant than benign testicular Leydig cell tumors, with co‐amplification being found in almost one‐third of malignant Leydig cell tumors, and none of the benign Leydig cell tumors [15]. Genomic profiling of metastatic testicular sex cord stromal tumors has identified potential therapeutic targets, including *CDK4* in Leydig cell tumors [16]. However, one must be cautious not to extrapolate findings in testicular Leydig cell tumors to AGCTs. Instead, these findings can serve as a harbinger of a potentially worthwhile area of investigation in AGCTs.

*ERBB3* (erb‐b2 receptor tyrosine kinase 3) encodes the HER3 protein, a member of the epidermal growth factor receptor (EGFR) family of receptor tyrosine kinases [17, 18]. Heterodimerization with other EGFR family members can lead to downstream cell proliferation or differentiation, and, according to the NIH gene database, its amplification has been implicated in numerous cancers. Its role in the pathogenesis of testicular AGCTs is not well characterized, but orthotopic mouse models have revealed high levels of ERBB family proteins in testicular germ cell tumors, with inhibition reducing tumoral testicular cell proliferation [17].

JAK2 dysregulation is well known to lead to increased cellular proliferation and predispose to myeloproliferative neoplasms of hematopoietic stem cells. The National Center for Biotechnology Information gene database shows that JAK2 is expressed at significant levels in the testes and ovaries, but we could not find any information regarding the clinical or prognostic significance of *JAK2* alterations in tumors [19].

The purpose of this case report is to add to the limited body of knowledge that we have concerning these fascinating and rare testicular tumors. There are less than 100 published cases of AGCTs with usable data concerning size, clinical presentation, and histological characteristics [1]. Each additional case will help bring us a step closer to uncovering key features and more accurate epidemiological data concerning this rare tumor.

## 4. Conclusion

We encountered a rare case of testicular AGCT in a 50‐year‐old African American male who presented to the Urology office with a month‐long history of right orchialgia and swelling. His history was notable for a right testicular lump present for 1–2 years and a right testicular cyst identified on ultrasound 7 years earlier. A right testicular ultrasound revealed a 2.0‐cm lesion along the upper to interpolar region with internal vascularity. The patient underwent right radical inguinal orchiectomy. The pathologic diagnosis was Granulosa Cell Tumor, Adult Type, pathologic stage classification pT1a, pN and pM not assigned. NGS showed low‐level amplification of *CDK4*, *MDM2*, *ERBB3*, and *JAK2*. No gene fusions or mutations in other genes were identified including the *FOXL2* p.C134W mutation that is characteristic for ovarian GCT. Follow‐up CT scans at 6 and 23 months post‐operation showed no evidence of metastatic disease in the chest, abdomen, or pelvis. At 42‐month follow‐up, the patient was disease‐free.

## Author Contributions

Christopher Moran, MD, Ruth Birbe, MD: writing – original draft.

Catherine Skibiel, MD, Hadi Shojaei, MD, Jeffrey J. Tomaszewski, MD, Tina Bocker Edmonston, MD, Veniamin Barshay, MD: writing – review and editing.

## Funding

This research received no external funding. Article publication charge was covered by Cooper Medical School of Rowan University.

## Disclosure

The authors have nothing to report.

## Ethics Statement

The approval of the research protocol by an institutional review board is not applicable for this study.

## Consent

Written informed consent was obtained from the patient for the publication of this case report and accompanying images.

## Conflicts of Interest

The authors declare no conflicts of interest.

## Data Availability

The data that support the findings of this study are available on request from the corresponding author. The data are not publicly available due to privacy or ethical restrictions.
